# Chemotherapeutic Nanoparticle-Based Liposomes Enhance the Efficiency of Mild Microwave Ablation in Hepatocellular Carcinoma Therapy

**DOI:** 10.3389/fphar.2020.00085

**Published:** 2020-02-26

**Authors:** Songsong Wu, Dongyun Zhang, Jie Yu, Jianping Dou, Xin Li, Mengjuan Mu, Ping Liang

**Affiliations:** ^1^ Department of Interventional Ultrasound, Chinese PLA General Hospital, Beijing, China; ^2^ Department of Ultrasonography, Fujian Provincial Hospital, Shengli Clinical Medical College of Fujian Medical University, Fuzhou, China

**Keywords:** hepatocellular carcinoma, liposomes, microwave ablation, combination therapy, enhance, nanoparticles

## Abstract

Hepatocellular carcinoma (HCC) is the third leading cause of death from cancer, and the 5-year overall survival (OS) rate for HCC remains unsatisfying worldwide. Microwave ablation (MWA) is a minimally invasive therapy that has made progress in treating HCC. However, HCC recurrence remains problematic. Therefore, combination therapy may offer better outcomes and enhance MWA efficiency through improved tumor control. We have developed doxorubicin-loaded liposomes (DNPs) as an efficient nanoplatform to enhance MWA of hepatocellular carcinoma even at the mild ablation condition. In this study, we demonstrated that the uptake of DNPs by HCC cells was increased 1.5-fold compared with that of free DOX. Enhanced synergism was observed in the combination of DNPs and MWA, which induced nearly 80% cell death. The combination of mild MWA and DNPs enhanced the ablation efficiency of HCC with significant inhibition of liver tumors and accounted for the longest survival rate among all groups. A much higher accumulation of the DNPs was observed in the transitional zone than in the ablation zone. No apparent systemic toxicity was observed for any of the treatments after 14 days. The present work demonstrates that DNPs combined with MWA could be a promising nanoparticle-based therapeutic approach for the treatment of hepatocellular carcinoma and shows potential for future clinical applications.

## Introduction

Hepatocellular carcinoma (HCC) ranks as the sixth most common malignancy worldwide and is the fourth leading cause of cancer death (Siegel et al., 2019). The 5-year overall survival (OS) rate for HCC remains poor worldwide (Costentin, 2017). Surgery is not always an option among patients with liver cancer as concurrent liver cirrhosis, which many of these patients have, increases the risk of mortality (Johnson and Wright, 2019). Minimally invasive therapies, such as radiofrequency ablation or microwave ablation (MWA) and high-intensity focused ultrasound ablation, offer new therapeutic approaches for HCC,(Zhu et al., 2018; Lyu et al., 2018; Mu et al., 2018; Huang et al., 2019) with the advantages of lower morbidity and mortality and broader indications compared with surgery (Shiina et al., 2018).

Rapid temperature, a larger ablation zone, and low susceptibility to the heat-sink effect are advantages that confer superiority to MWA for HCC therapy compared with other thermal modalities, (Facciorusso et al., 2016) particularly for early-stage HCC (Salati et al., 2017). These advantages overcome several challenges, including how the size of HCC can severely affect MWA efficacy, which may result in postsurgical recurrence or the development of unwanted lesions near important blood vessels and collateral biliary ducts that are difficult to ablate completely (Yu et al., 2017). High temperatures in the central zone near the antenna lead to necrosis and protein denaturation, whereas in the transitional zone, lower temperatures and the resulting energy insufficiency lead to a recurrence of more aggressive phenotypes and worse outcomes (Chu and Dupuy, 2014). Therefore, new strategies have been constantly researched.

Combination therapy may offer better outcomes to obtain an enhanced MWA efficiency with improved tumor control (Zhou et al., 2017). Previous studies have demonstrated that hyperthermia could augment chemotherapy, which in turn, enhanced ablation efficiency; therefore, temperature and chemotherapeutic agents acted to achieve tumor therapy (Yan et al., 2017). Nevertheless, liver cancer chemotherapy is not without its limitations, and the two major challenges are poor delivery efficiency and limited drug penetration into tumors (El Dika and Abou-Alfa, 2017). Nanoparticles as a drug delivery system has brought great improvements to cancer therapy (Dong et al., 2019). Nanoparticle therapeutics, based on natural or synthetic organic geo-macromolecules, have been developed as drug delivery systems by extending circulation times and facilitating uptake into tumors *via* enhanced permeability and retention (EPR) effect caused by leaky tumor vasculature (Yang et al., 2019). Liposomes were first approved by the US Food and Drug Administration and, since then, have attracted tremendous attention due to their high payload and easy synthesis, facilitating increased drug accumulation at tumor sites (Jha et al., 2016; Wang et al., 2019).

A combination therapy comprising ablation with chemotherapy offers several advantages over single ablation therapy, thereby decreasing ablation energy and enhancing tumor destruction (Fang et al., 2019; Xu et al., 2019). We therefore hypothesised that liposomes possessing optimal targeting properties and compatibility may transfer more necrosis-inducing drugs to the tumor and may thus be an effective chemical enhancer of MWA in HCC therapy. In this study, we used mild MWA energy, defined as small amounts of necrosis achieved through low microwave energy, to mimic the transitional zone. The literature on mild MWA combined with liposomes as an effective therapy to improve ablation outcomes and decrease recurrence is limited. We synthesised liposome-loading doxorubicin (DNPs), which enhanced the mild MWA effect and demonstrated that low microwave energy caused a relapse of all tumors, but a combination therapy could significantly reduce tumor growth and recurrence. Additionally, we provided evidence for the effectiveness of combination therapy in treating HCC or tumors located in difficult-to-access regions.

## Materials and Methods

### Reagents

Disaturated-phosphatidylcholine (DSPC) and 1,2 distearoyl-sn-glycero-3-phosphoethanolamine-N-[methoxy (poly-ethylene-glycol)-2000] (DSPE-PEG2000) were purchased from Avanti Polar Lipids, Inc. (USA). Cholesterol (Sigma-Aldrich, USA), DMEM and penicillin/streptomycin (HyClone, USA), fetal bovine serum (Biological Industries, Israel), DOX (Tokyo Chemical Industry, Japan), Gd-DTPA-HPDP (Consun, Guangzhou, China), RIPA Lysis Buffer (Beyotime, Shanghai, China), and a Cell Counting Kit-8 (CCK-8) (Dojindo Laboratories, Japan) were purchased and used as indicated by the manufacturer. Deionized water (18.2 MΩ cm) from a Milli-Q purification system was used for all reagent preparations.

### Synthesis and Characterisation of DNPs

The liposome formulation was composed of DSPC: cholesterol: DSPE-PEG-2000 dissolved in chloroform in a 55:40:5 molar ratio. The organic solvent was removed under nitrogen flow until a thin lipid film (10 mg) was formed. Then, the film was further dried for over 2 h under a vacuum. DOX was loaded *via* remote loading methods (Deng et al., 2014).The procedures were as follows: 1-ml (NH_4_)_2_SO_4_ solution (300 mM) was mixed with the lipid film and then hydrated *via* sonication at 65°C for 15 min at a frequency of 20 kHz and a power of 130 W for 6 min. The obtained rough liposomes were dialysed for 6 h with degassed water to remove the unloading (NH_4_)_2_SO_4_. Subsequently, the liposome suspension was mixed with 1.0-mg DOX and maintained at room temperature for 24 h. Finally, the particles were purified by centrifugation using 10-kDa molecular weight cutoff filters to remove unloading DOX. The dynamic light scattering (DLS) measurement of DNPs was performed using a Zetasizer (Malvern Nano ZS, UK). The Gd-DTPA-HPDP encapsulated DNPs were synthesised as mentioned above to capture images of the tumor uptake of DNPs. Liquids containing 1-ml (NH_4_)_2_SO_4_ solution and 5-mM Gd-DTPA-HPDP were mixed with the lipid film, and the mixture was sonicated and purified to obtain the encapsulated Gd-DTPA-HPDP DNPs.

### *In Vitro* Cellular Uptake and Ablation–Chemo Treatment

HepG2 and Huh7 cells were seeded into 24-well plates (3 × 10^5^/well), incubated with various concentrations of DNPs and DOX for 1, 3, and 6 h. Cells were washed with PBS to remove the drugs and were harvested for measurement of DOX fluorescence through flow cytometry (Beckman CytoFLEX, USA). Chemotherapeutic efficacy of DNPs was quantitatively evaluated by exposing HepG2 and Huh7 cells seeded into 96-well plates (1 × 10^5^/well) to various concentrations of DNPs and free DOX for 8 h. Cell viability was assessed using CCK-8 detection kits, and absorbance at 450 nm was measured using a multi-plate reader (Biotek, USA). All experiments were designed for three repeating groups.

To evaluate the combined cytotoxicity of DNPs with MWA, 1 × 10^4^ HepG2 cells were seeded into 96-well plates and incubated with 6.0 µg/ml DNPs and 6.0 µg/ml free DOX as a control in a 100 µl fresh medium. Cells were washed with PBS twice after an 8-h incubation and replaced with a 250-µl fresh medium. A microwave antenna (Kangyou Medical, China) was placed into the well, and cells were exposed to MWA (2450 MHz, 1.2 W) for 4 min. Cell viability was determined as described above. MWA followed the DNPs experiment and was conducted further to compare the outcome of chemoablation and ablation chemotherapy. Cells with a 250-µl medium were exposed to MWA (2450 MHz, 1.2 W) for 4 min. Then, cells were incubated with 6.0 µg/ml DNPs and 6.0 µg/ml DOX as a control in a 100 µl fresh medium. Eight hours later, cell viability was determined.

### Animal Experiments

Animals received care in accordance with the Guide for the Care and Use of Laboratory Animals published by the US National Institute of Health. The Chinese PLA General Hospital Animal Care and Use Committee approved all procedures employed in this study. Female BALB/c nude mice (6–8 weeks old) were maintained under aseptic conditions in a small animal isolator and were housed in groups of five in standard cages with access to food and water and a 12-h light/dark cycle. Animals were adapted to the animal facility for at least 7 days prior to the experiments. Mouse tumor models were established by subcutaneous injection of 2 × 10^6^ HepG2 cells in the right abdomen, and the tumor volume (mm^3^) was calculated as length × (width)^2^/2.

### Ablation–Chemo Combination Therapy in HCC Models

For tumor imaging, when the tumor size reached 10 × 10 mm, 100 µl of DNPs (100 µg/ml) was intravenously injected into tumor-bearing mice. An MRI examination (Bruker, Germany) was performed at various time points. To determine the efficiency of ablation–chemo, mice were divided into six groups (five per group), namely: (1) control group, (2) DOX group, (3) DNPs group, (4) MWA group, (5) MWA + DOX group, and (6) MWA + DNPs group. Mice were intravenously injected with 100 μl of PBS, DOX (100 μg/ml) or DNPs (containing 100 μg/ml of DOX), and 24 h later, mice were treated with MWA (2450 MHz, 1.0 W, 60 s). Thermal imaging was recorded through an infrared thermal imaging camera (Magnity Electronics Co., China). Three days later, intravenous injection of PBS, DOX, and DNPs were intravenously injected again, 24 h later, mice were treated with MWA, and the drug dose and MWA parameter were the same as in the first treatment. Changes in tumor volumes and body weight were recorded. Mice were sacrificed after 14 days, blood was collected for a biochemical panel evaluation (Mindary BC-5130, China), and tumors were collected for analysis. The survival experiments were repeated as aforementioned to calculate the survival of six groups. Considering the tumor burden of mice, a cutoff line was set, namely, a mouse burdened with a tumor volume larger than 1200 mm^3^ was considered dearth.

### Mild MWA Contributed to the Tumor Uptake of DNPs

HepG2 cells (1 × 10^4^) were seeded into 96-well plates and treated with a 250-µl fresh medium containing 6.0 µg/ml DNPs with a similar concentration of free DOX as the control. The microwave antenna was placed into the well, and cells were exposed to mild MWA at 1.0 W for 4 min. After an hour, cells were washed and examined with a microscope (Olympus BX43, Japan); at the same time, cells were lysed for quantitative analysis of DOX fluorescence using a multi-plate reader at an excitation wavelength 480 nm and emission wavelength 580 nm.

For *in vivo* experiments, when the tumor size reached 10 × 10 mm, 100 µl of DNPs (100 µg/ml) was intravenously injected into tumor-bearing mice. Eight hours later, mice were treated with MWA (2450 MHz, 1.0 W, 60 s). Eight hours after that, tumors were harvested from mice and imaged by microscope-based fluorescence imaging.

### *In Vivo* Toxicology Analysis

The blood of mice in the six groups on day 14 was collected for biochemical panel evaluation (Mindary BC-5130, China). Hepatic, heart, and kidney functional indicators of mice in the six groups on day 14 after treatment, such as alanine albumin (ALB), aspartate alkaline phosphatase aminotransferase (ALT), aminotransferase (AST), creatine kinase-MB (CK-MB), blood urine creatinine (Cr), blood urea nitrogen and urea, were tested to measure the responses of the experimental animals to the treatments.

### Statistical Analysis

The results are presented as the mean ± SD. Cell viability, tumor volume and weight, and mouse body weight were compared using a one-way ANOVA test and a Student’s t-test. A Student’s t-test was used to compare the blood biochemistry panel and complete blood counts between treatments. Differences were considered significant for p < 0.05.

## Results

### Synthesis and Cellular Uptake of DNPs and the Ablation–Chemo Effect

**Figure 1:**
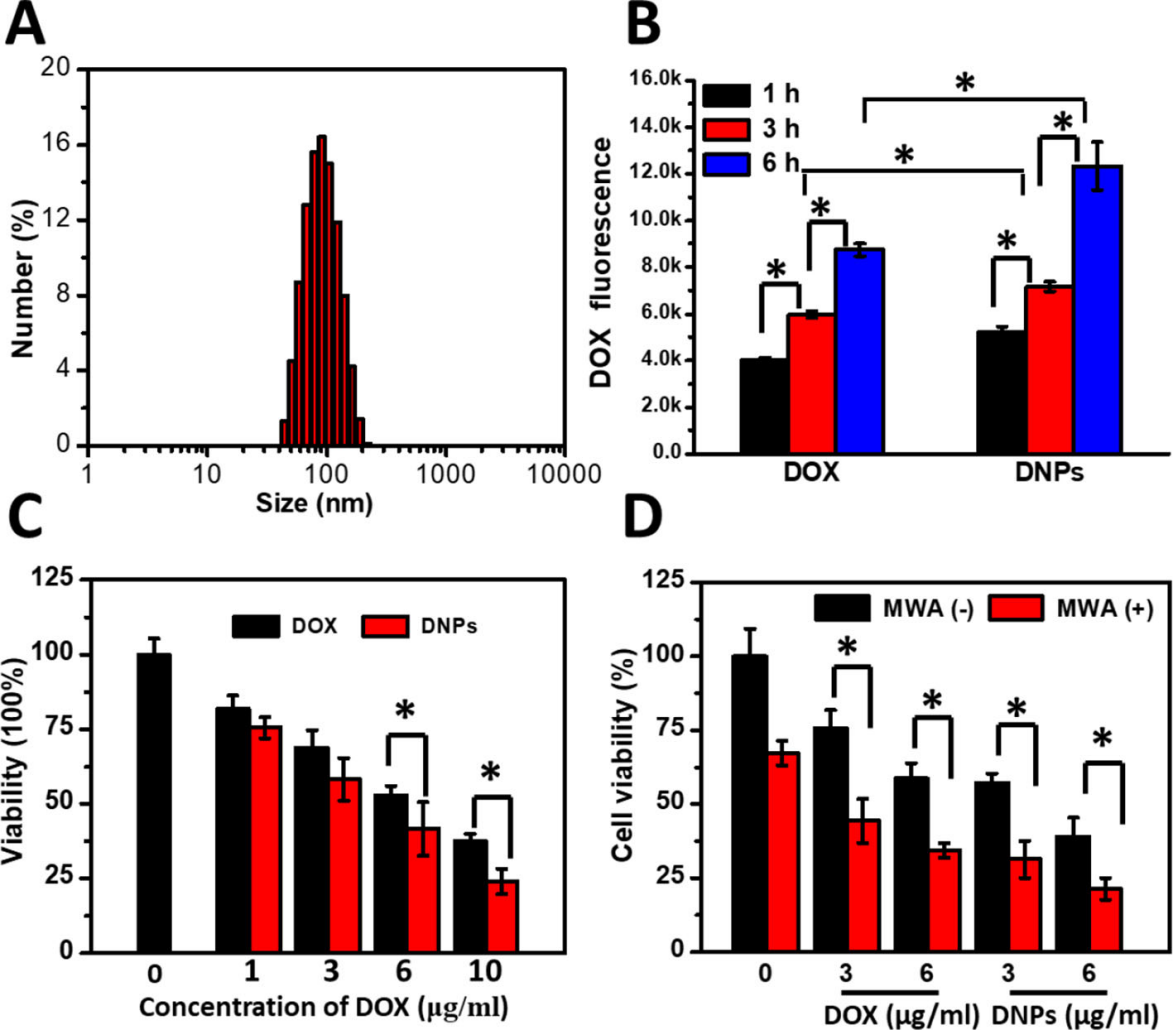
Characterization and cellular uptake of DNPs and the ablation-chemo effect. **(A)** DLS distribution of DNPs. **(B)** Intracellular DOX fluorescence of HepG2 treated with DNPs by flow cytometry. **(C)** Cell survival of cells treated with DOX and DNPs for 8 h. **(D)** Survival of cells treated with MWA at 1.2 W for 4 min after free DOX and DNPs at different concentrations incubated for 8 h. *p < 0.05.

The mixture of DSPC, cholesterol, and DSPE-PEG2000 at a molar ratio of 55:40:5 generated efficient encapsulation of DOX (>90%) into the liposomes through the remote loading method. DOX encapsulation achieved was at 94.3% ± 1.81%. The DLS results showed the hydrodynamic sizes of DNPs to measure 95.5 ± 4.32 nm with adequate size distribution (**Figure 1A**) and a negative charge (-20.13 ± 1.04 mV). Cellular uptake of DNPs by HCC cells was evaluated by flow cytometry, which showed that liposome-mediated cellular uptake generated significantly higher DOX fluorescence intensity compared with free DOX (p < 0.05) (**Figure 1B**). The difference was sustained over 1–6 h. The DOX uptake by DNPs increased 1.5-fold compared with that of free DOX (**Figure 1B**). The sensitivity of tumor cells to DNPs varied with the concentration when cell viability was tested using a standard Cell Counting Kit-8 (CCK-8) assay. The efficiency of cell death mediated by DNPs was positively correlated to the drug concentration and DOX encapsulation in liposomes showed better antitumor effects than DOX (**Figure 1C**). Cellular toxicity of DNPs was higher than free DOX, and cell viability of DNPs (41.65% ± 8.88%) was lower than that of DOX (52.77% ± 3.28%) at the concentration of 6.0 µg/ml, and the significance was obvious. Furthermore, another kind of human cancer cell, Huh7, was tested to validate the uptake and cytotoxicity experiments. The results showed that DNPs had higher fluorescence intensity compared with DOX (**Supporting S1**). The cytotoxicity of Huh7 was the same as the HepG2 result (**Supporting S2**).

To test whether MWA enhanced cellular toxicity mediated by DNPs, tumor cells were preincubated with DNPs and treated by MWA. MWA alone showed little cellular suppression at 1.2 W/cm^2^ for 4 min, and cell viability was 67.3% ± 4.19% (**Figure 1D**). For the DNPs group, the viabilities were 57.15% ± 3.24% and 39.15% ± 6.24% at 3 μg/ml and 6 μg/ml, respectively. However, the combination of DOX and MWA enhanced cell death and the cell viability were 44.41% ± 7.52% and 34.41% ± 2.52% at 3 μg/ml and 6 μg/ml, respectively. An enhanced effect was observed with the combination of DNPs and MWA, the cell viability of 31.31% ± 6.18% and 21.31% ± 3.68% at 3 μg/ml and 6 μg/ml, respectively. Thus, chemoablation of DNPs exerted higher cellular toxicity than DOX, which is in accordance with the results of the cellular cytotoxicity test. In addition, we further tested MWA followed by DNPs. The results showed that DNPs followed by MWA had less viability than MWA followed by DNPs (**Supporting S3**). These experiments illustrated that chemoablation obtained better outcomes than ablation followed by chemotherapy.

### *In Vivo* Imaging of DNPs in HCC by MRI

**Figure 2:**
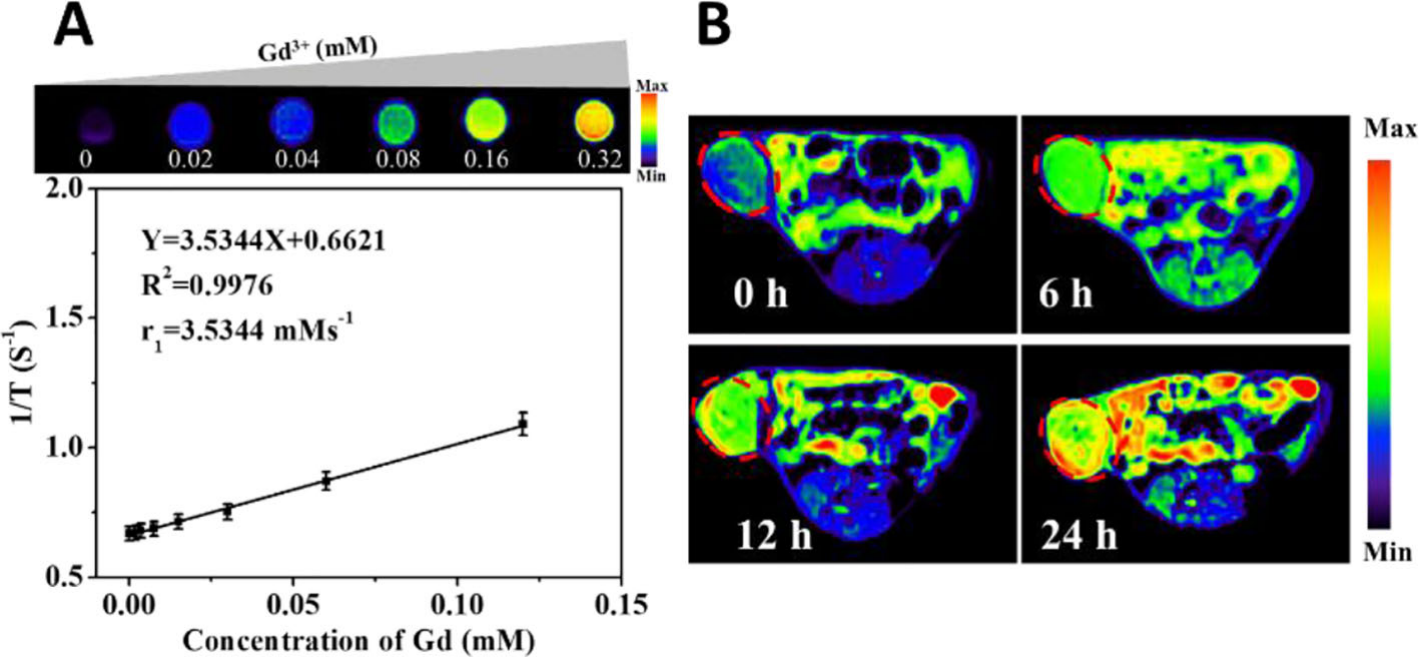
MRI images of Gd-DTPA-HPDP encapsulated DNPs *in vivo*. **(A)** Quantitative curve of the MRI intensity of DNPs at different concentrations. **(B)** MRI images of mice at different time points after DNPs injection.

Gd-DTPA-HPDP encapsulated DNPs as MRI contrast agents can be easily tracked to visualise the drug delivery process through the measurement of T1 relaxation and various concentrations of liposomes. The corresponding quantitative analysis revealed that the MRI signal correlated linearly to the concentration of DNPs from 0 to 0.32 µM of Ga^3+^ (**Figure 2A**). An *in vivo* MRI scan was performed to investigate the accumulation of DNPs in tumors, and it showed a gradual increase in the MRI intensity of DNPs in the tumor, and over a 24 h continuous observation period, a sufficient amount of DNPs efficiently and passively targeted the tumor site (**Figure 2B**).

### Ablation Chemotherapy in HCC Models

**Figure 3:**
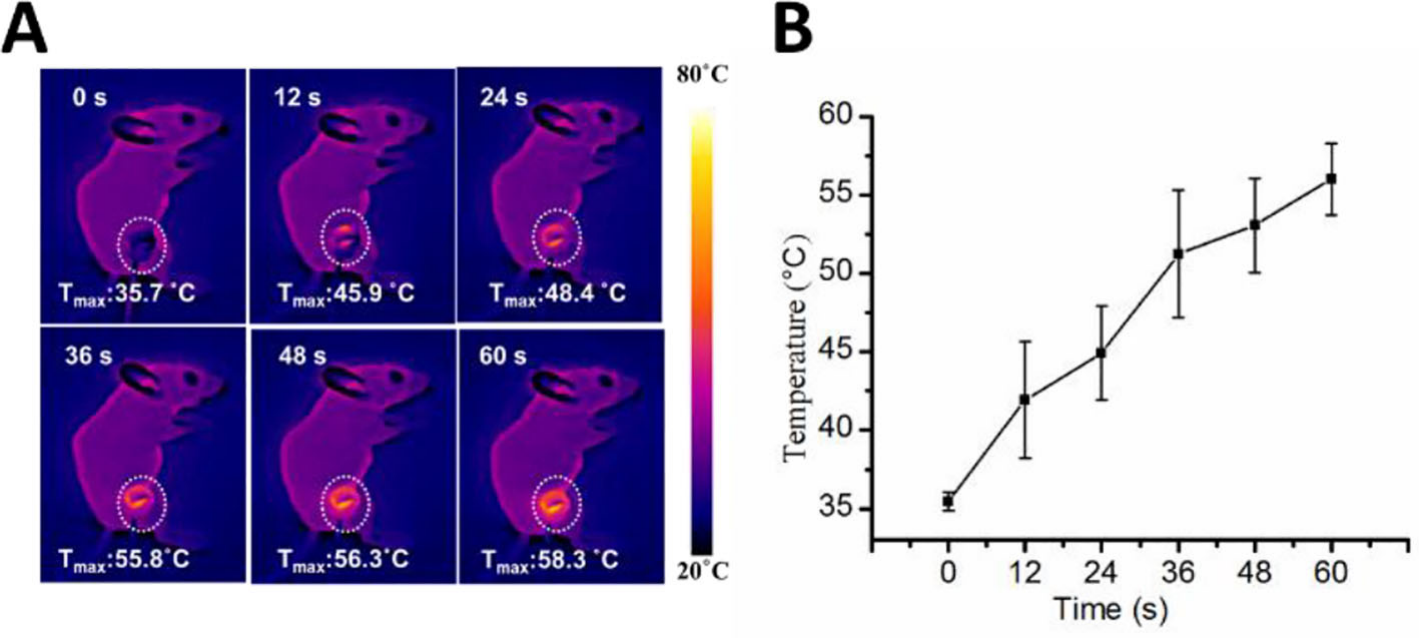
Infrared thermo-graphic maps of the MWA process. **(A)** Infrared thermo-graphic maps of mice exposed to MWA for 1min at 1.2 W/cm^2^. **(B)** Maximum temperature profiles of MWA after injection of DNPs after 24 h.

**Figure 4:**
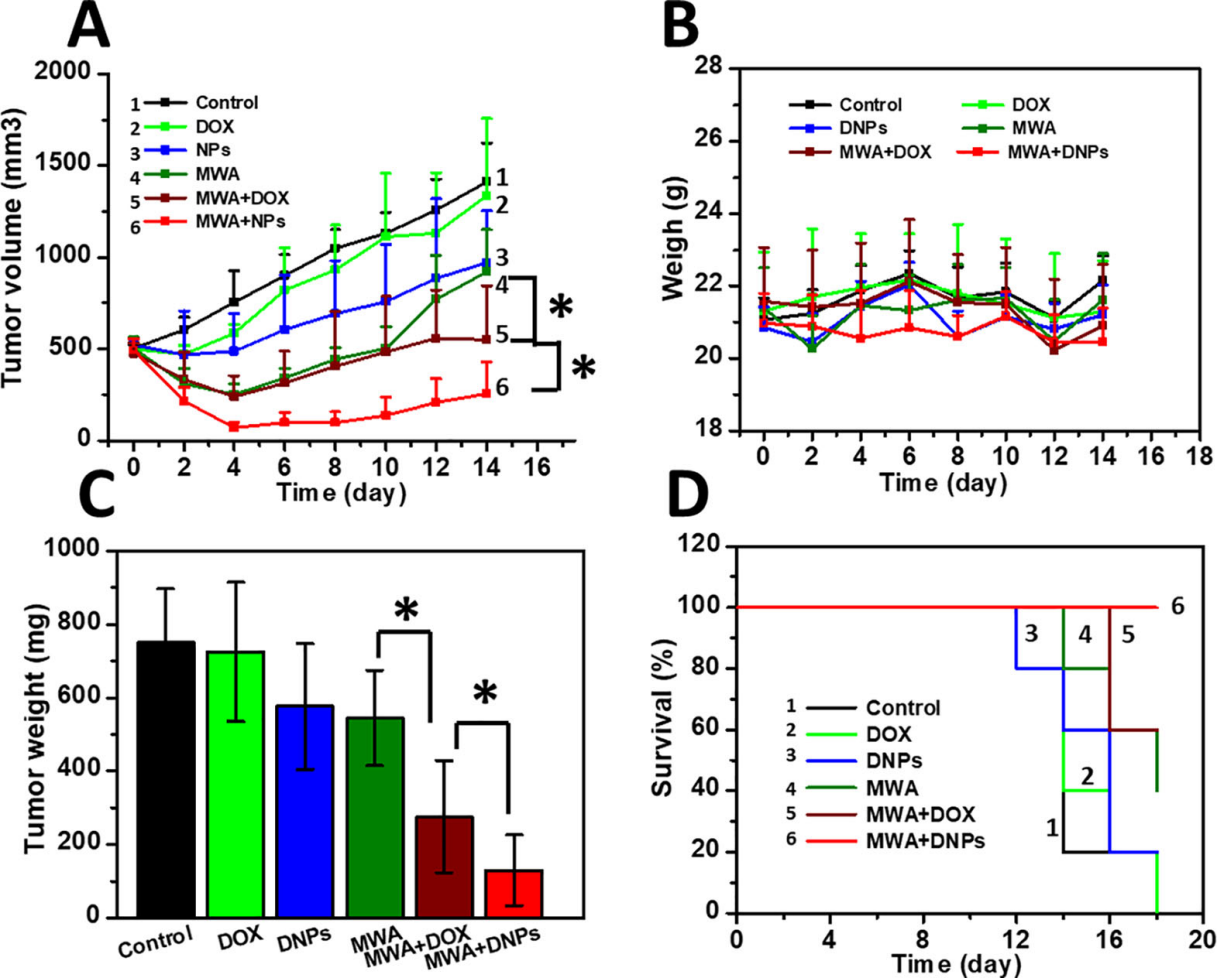
Antitumor effect in HCC models. **(A)** Tumor growth curve of six mice groups after mild MWA treatment. **(B)** Survival curve of mice of six groups after mild MWA treatment. **(C)** tumor weights of all mice after being sacrificed on day 14. **(D)** Body weight change of mice curve for 14 days. *p < 0.05.

We measured the intra-tumoral temperature when MWA was carried out by 1 W/cm^2^ for 60 s. The DNPs had a maximum temperature at 55.8°C, 56.3°C, and 58.3°C at 36, 48, and 60 s, respectively (**Figures 3A, B**). The maximum temperature of both the MWA and MWA + DOX group did not differ compared with the MWA + DNPs group (data not shown). Investigation of the antitumor properties of chemoablation in HCC models showed that tumors treated with DOX were not significantly different from the control group (1334.68 ± 420.15 mm^3^ vs 1411.21 ± 209.71 mm^3^) (**Figure 4A**). However, in the DNPs group, inhibition of tumor growth was significantly higher than that of the DOX group (969.37 ± 284.08 mm^3^ vs 1411.21 ± 209.71 mm^3^). Additionally, combination therapy of DNPs plus MWA injected twice led to a satisfactory tumor remission (256.44 ± 172.35 mm^3^). It was noted that MWA and MWA + DOX inhibited tumor growth less effectively than MWA + DNPs, which was attributed to the ERP effect of DNPs (**Figure 4B**). Furthermore, the OS duration of mice treated with MWA, MWA + DOX, and MWA + DNPs significantly increased when compared with the control group. Survival of MWA + DNPs group was 100% on day 14.

On day 14 after MWA, the mean tumor weights were 750.46 ± 144.58 mg, 724.18 ± 189.99 mg, and 576.04 ± 170.4 mg for the control, DOX, and DNPs, respectively (**Figure 4C**). The reduction in tumor weight in the DNPs group confirmed that DNPs exerted a significant anticancer effect in HCC, owing to accumulated drug cytotoxicity. On the other hand, the mean tumor weights of MWA, MWA + DOX, and MWA + DNPs were 543.46 ± 129.64 mg, 275.98 ± 152.96 mg, and 129.7 ± 96.88 mg, respectively (**Figure 4C**), which shows that MWA treatment could significantly affect tumor growth. Since the weight curve analysis showed no body weight loss during the observation period in all groups, there was no apparent systemic toxicity observed for any of the treatments after 14 days (**Figure 4D**).

### Mild MWA Promoted Tumor Uptake of DNPs

**Figure 5:**
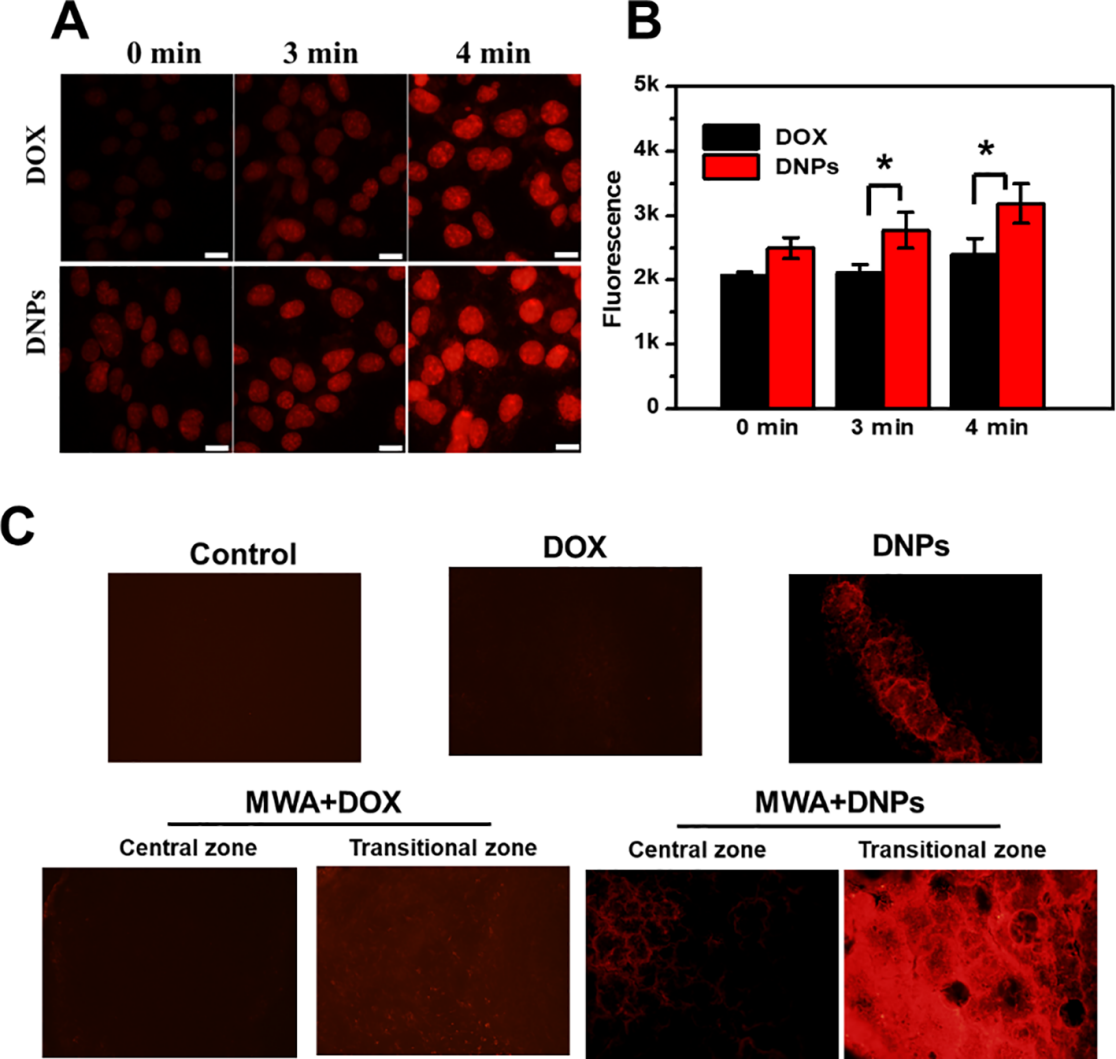
Mild microwave ablation enhanced the tumor uptake of DNPs. **(A)** Cellular uptake of DNPs under mild MWA. **(B)** Quantitative cellular uptake of DOX and DNPs after mild MWA with multiplate reader. **(C)** Images of tumor tissue uptake of DNPs after mild MWA. *p < 0.05.

Uptake of DNPs into the tumor and cells under mild MWA to mimic the transitional MWA condition showed increased tumor uptake of DNPs after MWA treatment. As shown in **Figure 5A**, an increase in fluorescence of DNPs with MWA duration was observed as fluorescence at 4 min after MWA was more intense than that at 3 min. This was true for both DOX and DNPs. DOX fluorescence was significantly lower than that of the DNPs, and quantitative analysis revealed that the cellular uptake of DNPs was increased 1.1-fold over DOX after mild MWA treatment for 4 min (**Figure 5B**). The cellular uptake of DNPs was significantly higher than that of DOX, as shown in the *in vivo* experiments (**Figure 5C**), where the DNPs in the transitional zone were much higher than those in the ablation zone.

### *In Vivo* Toxicology Analysis

**Figure 6:**
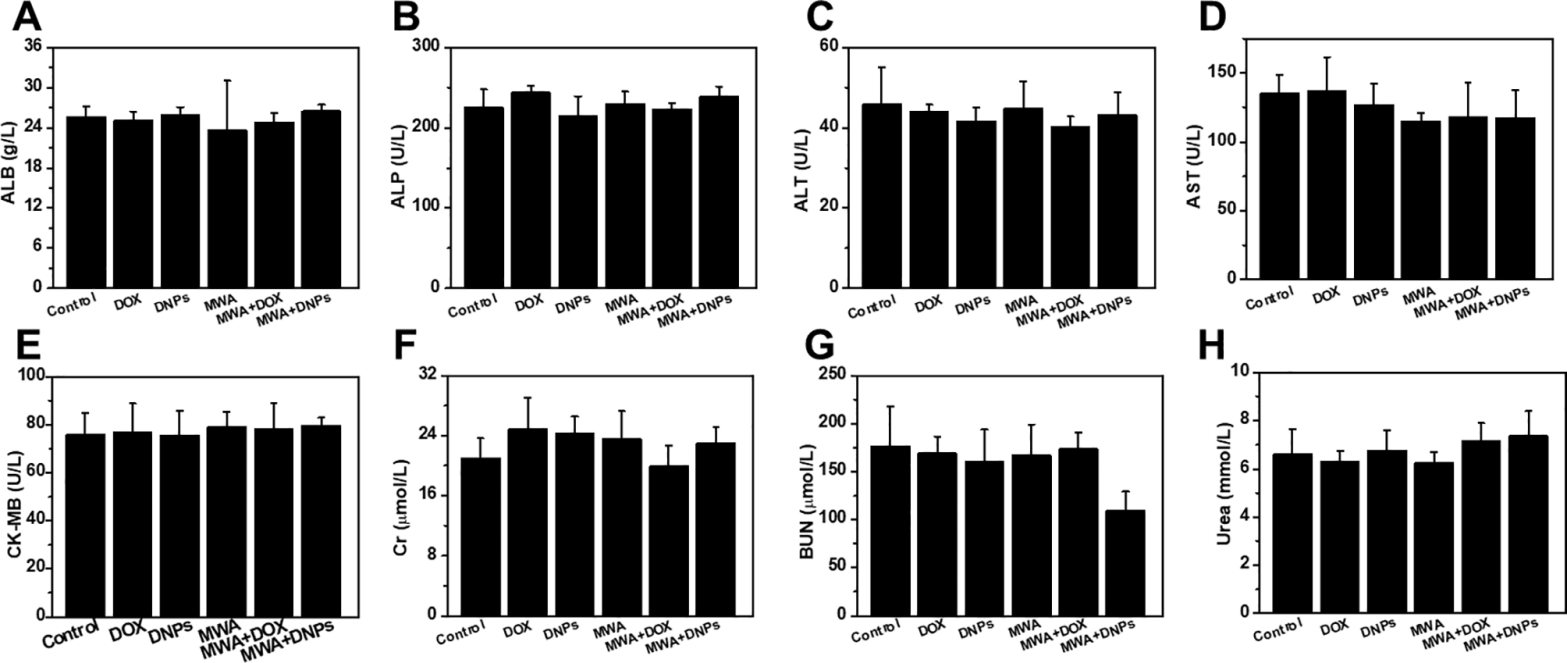
Hepatic, heart, and kidney functional indicators of mice with different treatment. **(A)** ALB, **(B)** ALP, **(C)** ALT, **(D)** AST, **(E)** CK-MB, **(F)** Cr,**(G)** UN, **(H)** UREA vis blood biochemistry test.

Toxicity associated with each treatment was investigated *via in vivo* blood biochemistry tests, and as shown in **Figures 6A−H**, MWA, DOX, DNPs, MWA + DOX, and MWA + DNPs showed no significant differences with the control group in the levels of these markers, indicating the favourable hepatic, heart, and renal safety profile of MWA, DOX, DNPs, MWA + DOX, and MWA + DNPs in mice.

## Discussion

There have been tremendous improvements in HCC therapy during the last two decades, especially in the use of minimal interventional therapies (Facciorusso et al., 2016; Salati et al., 2017; Shiina et al., 2018). Microwave ablation is one such method that has shown promising outcomes for HCC treatment, due to the rapid temperature increase and larger ablation compared with other modalities (Zhang et al., 2019). Despite being a promising technique for HCC ablation, MWA faces some challenges, including the limited ablation zone and the thermal injury risk for adjacent visceral tissues (Facciorusso et al., 2016). Since HCC tumors often recur after MWA, preventing or reducing HCC relapse after MWA is a major clinical challenge. However, we applied a combination therapy, which conferred more benefits than MWA alone, particularly for HCC or tumors adjacent to the gastrointestinal tract to surmount those challenges (Zhou et al., 2017).

The EPR effect endows nanoparticles with unique advantages over chemotherapy (Chen et al., 2018). In this study, we designed DNPs as an effective nanomedicine when combined with MWA to improve the outcome of HCC therapy. Intracellular DOX concentration of DNPs was higher than DOX by liposome-mediated cellular uptake, which led to higher cytotoxicity of DNPs than DOX. Chemotherapy in HCC often achieves underwhelming anticancer effects, and an urgent need for alternative approaches led to a combination therapy to treat HCC. The combination of ablation and nanoparticle-mediated chemotherapy has been demonstrated in several studies to significantly improve tumor control rates and prolong survival (Yang et al., 2012; Wang et al., 2016). We provided evidence in this study for the combination of MWA + DNPs to significantly suppress *in vivo* tumor growth compared with single treatment with DNPs and MWA, indicating that the combined effect was achieved. Our results suggest that the suppression mediated by MWA could be boosted by combination therapy. Thus, it is plausible that ablation with DNPs exceeded the outcome of MWA and increased its potency. Thus, combination therapy showed better antitumor properties than MWA alone.

Since tumor volume and weight in the MWA + DNPs group were significantly lower than that of other groups, it is plausible that the tumors were completely eradicated during treatment. For tumors larger than 5–10 cm, insufficient ablation of the periphery of the tumor often led to recurrent HCC. Therefore, in this study, we used mild MWA energy to mimic the transitional zone, where the resulting mild hyperthermia in the transitional zone increased tumor temperatures to 42°C, which could not kill all tumor cells (Chu and Dupuy, 2014). However, mild hyperthermia and its secondary effects may be directly lethal to some cancer cells and sensitise cells to chemotherapy (Solazzo et al., 2010). The tumor vascellums could be reconstructed by the thermal effect, and enhanced tumor uptake has been observed in previous photodynamic therapies. In our study, DNPs accumulated more in the transitional zone than in the ablation zone, which led to extravasation of nanoparticles at the site, (Zhen et al., 2014) resulting in a lethal effect on the tumors and an improvement in the outcome of MWA. This could be attributed to the open vasculature in the transitional zone that increased the accumulation of DNPs in the tumor. Our findings are robust and support the notion that further development of chemotherapeutic nanomedicine combined with MWA presents a promising and effective treatment strategy for HCC due to its optimal tumor-targeting properties and enhanced drug delivery efficacy.

## Conclusion

In the present study, we described the development of DNPs, a nanoplatform comprising liposomes loaded with DOX that robustly enhance mild MWA therapy in HCC, indicating a substantial antitumor efficacy. Chemoablation therapy in the HCC model used in this study led to improved survival rates by enhancing the precision and efficiency of the targeted approach adopted. Thus, DNPs hold considerable promise for clinical HCC therapy as a result of its favourable HCC targeting properties.

## Data Availability Statement

All datasets generated for this study are included in the article/**Supplementary Material**.

## Ethics Statement

The animal study was reviewed and approved by the Chinese PLA General Hospital Animal Care and Use Committee.

## Author Contributions

PL designed the study. SW, DZ, JD, XL, and MM carried out the experiments. JY revised the manuscript. SW and PL wrote the manuscript.

## Conflict of Interest

The authors declare that the research was conducted in the absence of any commercial or financial relationships that could be construed as a potential conflict of interest.
