# Supplementary material for: Chemotherapeutic Nanoparticle-Based Liposomes Enhance the Efficiency of Mild Microwave Ablation in Hepatocellular Carcinoma Therapy
Source: Front Pharmacol. 2020 Feb 26;11:85. doi: 10.3389/fphar.2020.00085 (PMC7054279; doi:10.3389/fphar.2020.00085)
Supplement: Supplementary file 1 [file DataSheet_1.docx]

**Chemotherapeutic nanoparticle-based liposomes enhance the efficiency of mild microwave ablation in hepatocellular carcinoma therapy**

Songsong Wu^1,2^, Dongyun Zhang ^1^, Jie Yu^1^, Jianping Dou^1^, Xin Li1, Mengjuan Mu^1^, Ping Liang^1*^

Authors affiliation

1. Department of Interventional Ultrasound, Chinese PLA General Hospital, 28 Fuxing Road, Beijing 100853, China.

2. Department of Ultrasonography, Fujian Provincial Hospital, Shengli Clinical Medical College of Fujian Medical University, Fuzhou 350001, China

*Contact information:

Ping Liang, M.D., Ph.D., Department of Interventional Ultrasound, Chinese PLA General Hospital, 28 Fuxing Road, Beijing 100853, China. Fax: +86-10-68161218; E-mail: liangping301@hotmail.com.


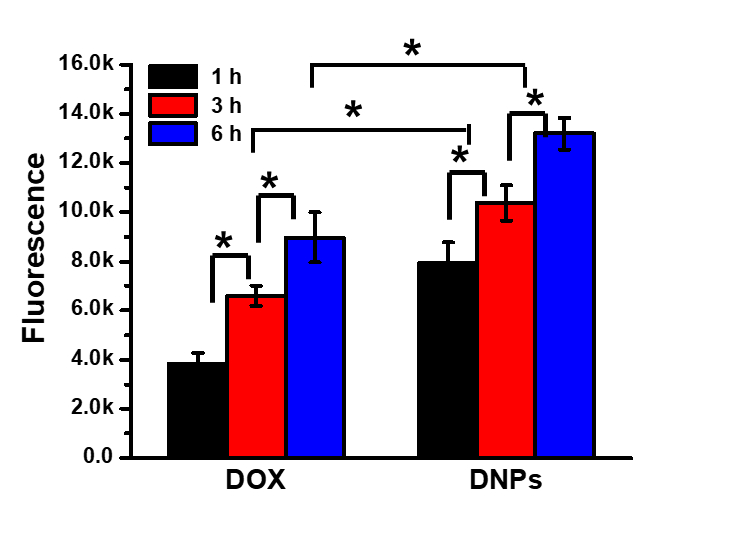


Supporting S1: Intracellular DOX fluorescence of Huh7 cells treated with DNPs by flow cytometry.


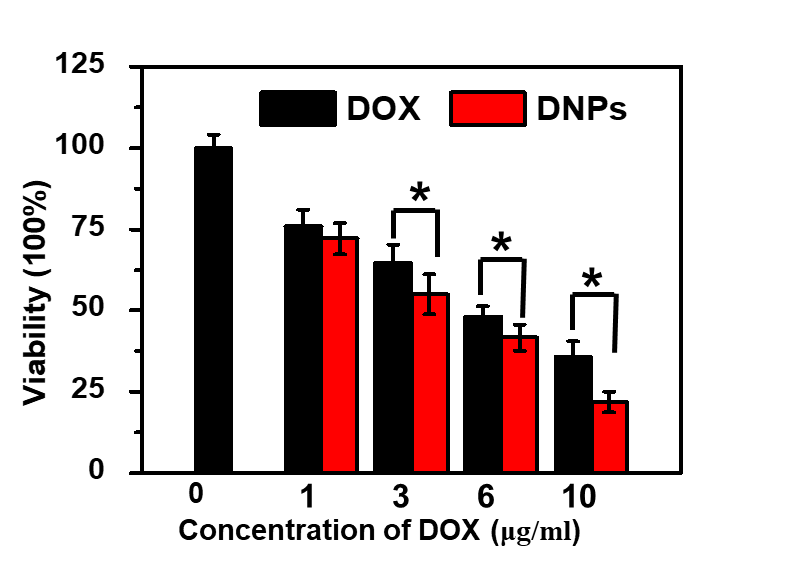


Supporting S2: Cell survival of Huh7 cells treated with DOX and DNPs.

Supporting S3: Survival of cells treated with MWA followed DOX and DNPs incubation.
